# Supplementary material for: Comparing quantile regression spline analyses and supervised machine learning for environmental quality assessment at coastal marine aquaculture installations
Source: PeerJ. 2023 Jun 13;11:e15425. doi: 10.7717/peerj.15425 (PMC10274583; doi:10.7717/peerj.15425)
Supplement: Supplemental Information 1 — The classification based on Phillips et al. (2014) is as follows: IQI 0–0.24 = High status, IQI 0.25–0.43 =Good status, IQI 0.44–0.63 = Moderate status, IQI 0.64–0.74 = Poor status, IQI 0.75–1 = Bad status. [file peerj-11-15425-s001.pdf]

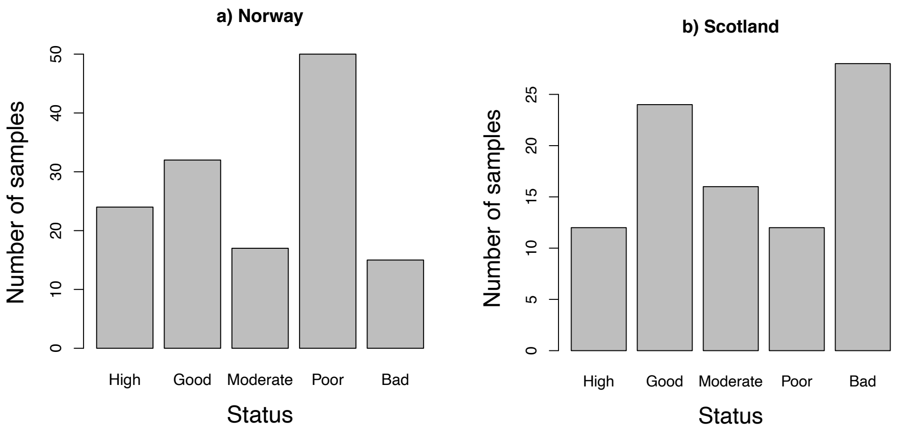

Figure S1 The environmental status of samples based on IQI quality intervals for a) Norwegian samples (n= 138) and b) Scottish samples (n=92). The classification based on Phillips et al. (2014) is as follows: IQI 0-0.24 = High status, IQI 0.25-0.43 =Good status, IQI 0.44-0.63 = Moderate status, IQI 0.64-0.74 = Poor status, IQI 0.75-1 = Bad status.
