# Supplementary material for: Comparing quantile regression spline analyses and supervised machine learning for environmental quality assessment at coastal marine aquaculture installations
Source: PeerJ. 2023 Jun 13;11:e15425. doi: 10.7717/peerj.15425 (PMC10274583; doi:10.7717/peerj.15425)
Supplement: Supplemental Information 2 [file peerj-11-15425-s002.pdf]

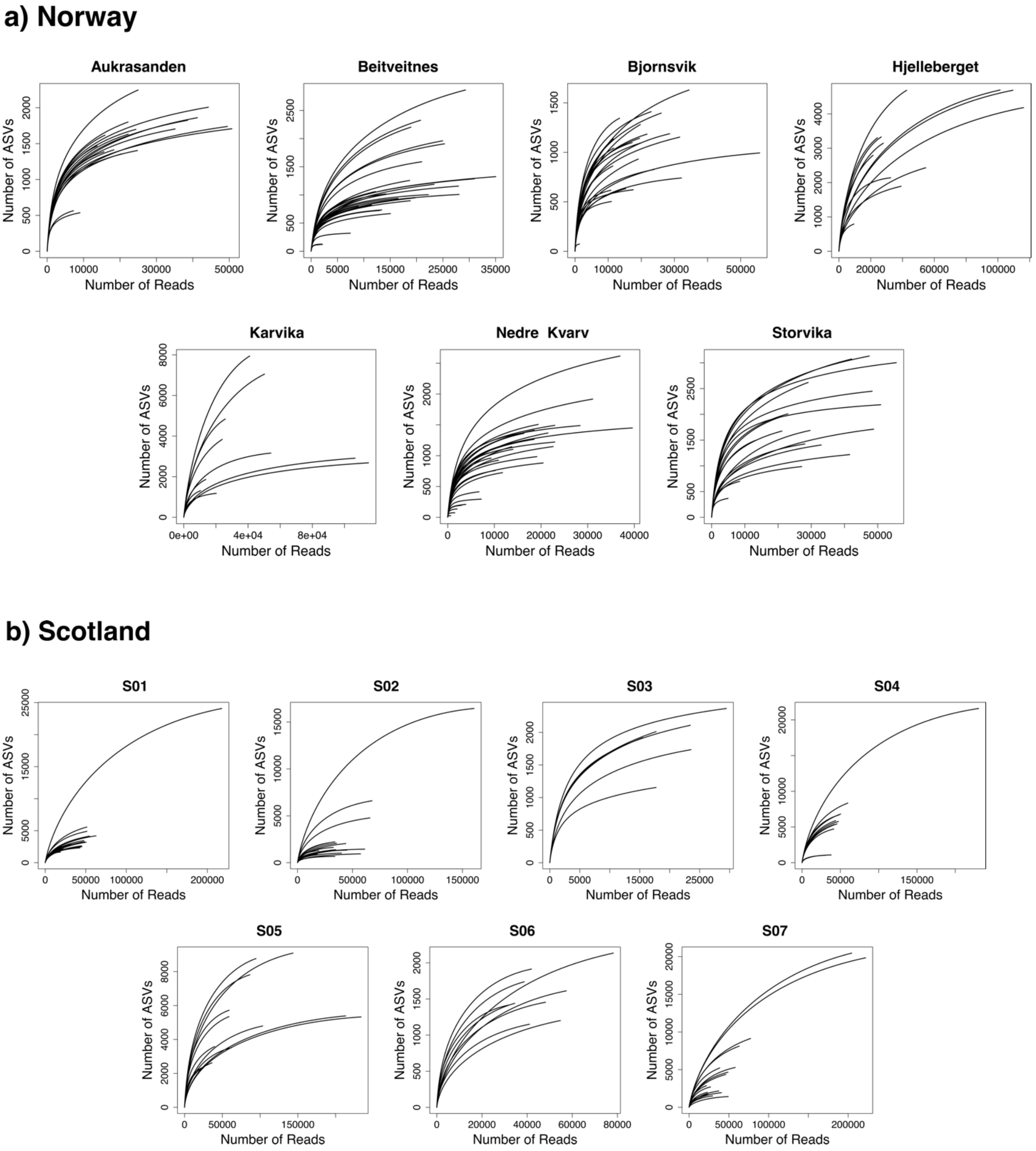

Figure S2 Rarefaction curves showing the number of sequence reads against the number of ASVs for a) Norwegian farms (n= 138 samples) and b) Scotland (n=92 samples).
