# Supplementary material for: Comparing quantile regression spline analyses and supervised machine learning for environmental quality assessment at coastal marine aquaculture installations
Source: PeerJ. 2023 Jun 13;11:e15425. doi: 10.7717/peerj.15425 (PMC10274583; doi:10.7717/peerj.15425)
Supplement: Supplemental Information 3 — The ecological quality of the samples is indicated by a color scheme, ranging from good environmental quality (high IQI, in dark blue) to a low environmental quality (in light blue, low IQI). NMDS is based on Bray-Curtis dissimilarity. In the left panels, the dissimilarity of samples based on all ASVs with at least 0.001% contribution to all reads is shown, corresponding to 9,132 ASVs for the Norwegian dataset and 8,663 ASVs for the Scottish dataset. The right panels are based on the most abundant 250 ASVs per dataset which were used for downstream analysis. [file peerj-11-15425-s003.pdf]

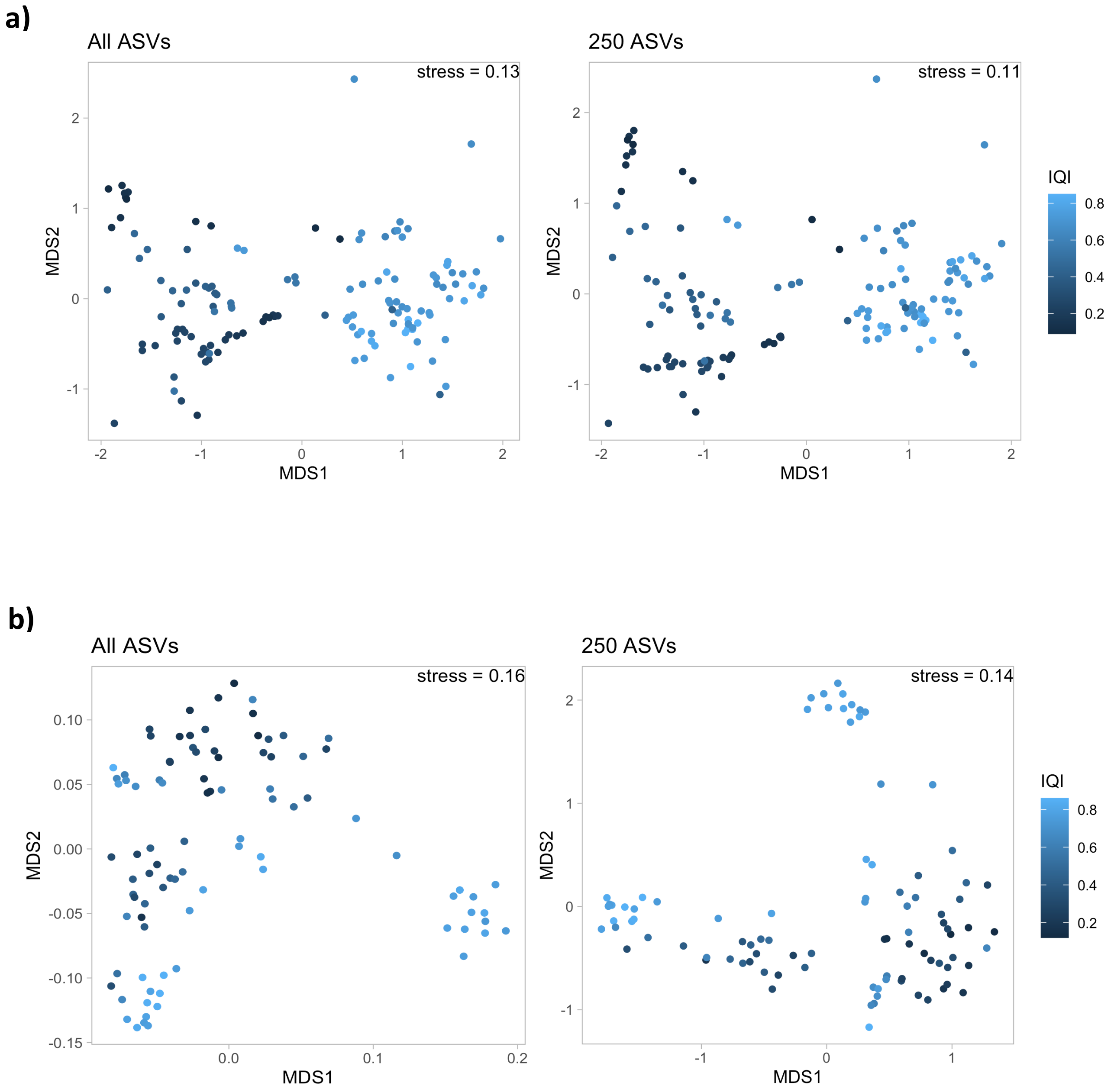

Figure S4 NMDS of samples for a) Norwegian salmon farm sediment and b) Scottish salmon farm sediement. The ecological quality of the samples is indicated by a color scheme, ranging from good environmental quality (high IQI, in dark blue) to a low environmental quality (in light blue, low IQI). NMDS is based on Bray-Curtis dissimilarity. In the left panels, the dissimilarity of samples based on all ASVs with at least 0.001% contribution to all reads is shown, corresponding to 9132 ASVs for the Norwegian dataset and 8663 ASVs for the Scottish dataset. The right panels are based on the most abundant 250 ASVs per dataset which were used for downstream analysis.
