# Supplementary material for: Comparing quantile regression spline analyses and supervised machine learning for environmental quality assessment at coastal marine aquaculture installations
Source: PeerJ. 2023 Jun 13;11:e15425. doi: 10.7717/peerj.15425 (PMC10274583; doi:10.7717/peerj.15425)
Supplement: Supplemental Information 4 — Quantile regression splines were constructed at the 95th percentile (df = 3). The IQI value at which the ASV has its peak abundance within a farm is indicated with a vertical line. [file peerj-11-15425-s004.pdf]

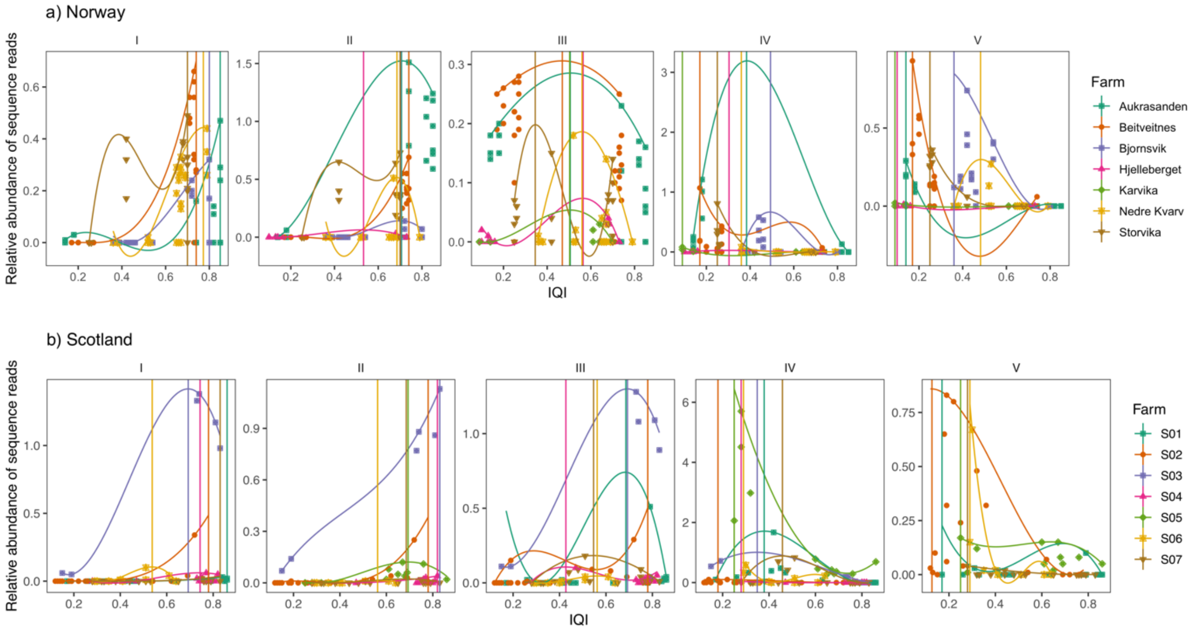

Figure S3 Quantile Regression Spline plots showing the relative abundance of sequences reads of selected bacterial ASVs from Eco-Groups I to V as a function to the Infaunal Quality Index (IQI) for a) Norway salmon farms and b) Scotland salmon farms. Quantile regression splines were constructed at the 95th percentile (df = 3). The IQI value at which the ASV has its peak abundance within a farm is indicated with a vertical line.
