# Supplementary material for: Comparing quantile regression spline analyses and supervised machine learning for environmental quality assessment at coastal marine aquaculture installations
Source: PeerJ. 2023 Jun 13;11:e15425. doi: 10.7717/peerj.15425 (PMC10274583; doi:10.7717/peerj.15425)
Supplement: Supplemental Information 6 — With bold are indicated the ASVs that were in the top 20 ASVs with the highest importance value by Random Forest (RF). [file peerj-11-15425-s006.docx]

**Table S2** List of indicators for a) Norway and b) Scotland datasets sorted by relative abundance and their assigned taxonomic affiliation and Eco-group. With bold are indicated the ASVs that were in the top 20 ASVs with the highest importance value by Random Forest (RF).

1. Norway

| ASV | Taxa name | Eco-group | Relative abundance |
| --- | --- | --- | --- |
| **ASV_000031** | **Myxococcales** | **III** | **1.03** |
| **ASV_000024** | **Alteromonadales** | **IV** | **0.92** |
| ASV_000043 | Myxococcales | III | 0.90 |
| **ASV_000040** | **Myxococcales** | **II** | **0.86** |
| ASV_000016 | Helicobacteraceae | IV | 0.83 |
| **ASV_000013** | **Helicobacteraceae** | **IV** | **0.72** |
| ASV_000022 | Helicobacteraceae | IV | 0.54 |
| ASV_000100 | *Psychromonas* | IV | 0.51 |
| **ASV_000054** | **Syntrophobacteraceae** | **II** | **0.49** |
| ASV_000039 | Bacteroidales | IV | 0.48 |
| ASV_000038 | Thiohalorhabdales | IV | 0.46 |
| **ASV_000036** | **Bacteroidales** | **IV** | **0.46** |
| **ASV_000063** | **Rhodospirillales** | **II** | **0.45** |
| ASV_000055 | Alteromonadales | IV | 0.41 |
| **ASV_000126** | ***Nitrospina*** | **II** | **0.40** |
| ASV_000076 | Desulfobulbaceae | IV | 0.36 |
| ASV_000106 | Myxococcales | III | 0.36 |
| **ASV_000044** | **Flavobacteriaceae** | **IV** | **0.35** |
| ASV_000052 | Helicobacteraceae | IV | 0.30 |
| ASV_000114 | *Desulfococcus* | II | 0.29 |
| ASV_000026 | Acidimicrobiales | IV | 0.27 |
| ASV_000066 | Caldilineaceae | IV | 0.27 |
| ASV_000173 | *Psychromonas* | IV | 0.25 |
| ASV_000046 | Helicobacteraceae | IV | 0.25 |
| ASV_000233 | Myxococcales | I | 0.24 |
| **ASV_000198** | **Gammaproteobacteria** | **II** | **0.23** |
| ASV_000086 | Bacteroidales | IV | 0.22 |
| ASV_000186 | Nitrospiraceae | II | 0.22 |
| ASV_000155 | Myxococcales | III | 0.22 |
| ASV_000033 | Enterobacteriaceae | III | 0.22 |
| ASV_000085 | Flavobacteriaceae | IV | 0.20 |
| ASV_000128 | Unknown bacteria | IV | 0.20 |
| ASV_000098 | Caldilineaceae | IV | 0.20 |
| ASV_000149 | Myxococcales | II | 0.20 |
| ASV_000123 | Alteromonadales | IV | 0.19 |
| ASV_000090 | Thiohalorhabdales | IV | 0.19 |
| ASV_000238 | Piscirickettsiaceae | III | 0.18 |
| ASV_000153 | Lachnospiraceae | IV | 0.17 |
| ASV_000014 | Helicobacteraceae | IV | 0.17 |
| ASV_000271 | Myxococcales | III | 0.17 |
| **ASV_000296** | **Helicobacteraceae** | **IV** | **0.17** |
| **ASV_000080** | ***Desulfosarcina*** | **IV** | **0.16** |
| ASV_000268 | *Psychromonas* | IV | 0.16 |
| ASV_000208 | *Psychromonas* | IV | 0.16 |
| ASV_000276 | Gammaproteobacteria | II | 0.16 |
| ASV_000165 | Desulfobacteraceae | IV | 0.16 |
| ASV_000134 | Ruminococcaceae | IV | 0.16 |
| ASV_000385 | Piscirickettsiaceae | II | 0.16 |
| ASV_000273 | Myxococcales | II | 0.14 |
| ASV_000456 | Acidimicrobiales | II | 0.14 |
| ASV_000377 | Piscirickettsiaceae | II | 0.14 |
| ASV_000357 | Nitrospiraceae | II | 0.14 |
| ASV_000437 | Alphaproteobacteria | II | 0.14 |
| **ASV_000217** | **Acidobacteria** | **III** | **0.14** |
| **ASV_000053** | ***Lutimonas*** | **IV** | **0.12** |
| ASV_000214 | Desulfarculaceae | IV | 0.12 |
| ASV_000776 | Alteromonadales | II | 0.12 |
| ASV_000488 | Myxococcales | I | 0.11 |
| ASV_000420 | Gammaproteobacteria | II | 0.11 |
| ASV_000472 | Gammaproteobacteria | II | 0.11 |
| **ASV_000348** | **Bacteroidales** | **IV** | **0.11** |
| ASV_000422 | Betaproteobacteria | I | 0.11 |
| ASV_000243 | Chromatiales | III | 0.11 |
| ASV_000281 | Desulfobacteraceae | IV | 0.11 |
| ASV_000486 | Piscirickettsiaceae | II | 0.10 |
| ASV_000050 | Helicobacteraceae | IV | 0.10 |
| ASV_000171 | Gammaproteobacteria | IV | 0.10 |
| ASV_000494 | Gemmatimonadetes | II | 0.10 |
| ASV_000275 | Desulfobulbaceae | IV | 0.10 |
| ASV_000438 | Flammeovirgaceae | III | 0.10 |
| ASV_000662 | Piscirickettsiaceae | II | 0.10 |
| ASV_001328 | Piscirickettsiaceae | II | 0.09 |
| ASV_000064 | Lutimonas | IV | 0.09 |
| ASV_000325 | Alteromonadales | II | 0.09 |
| ASV_000323 | Verrucomicrobia | IV | 0.09 |
| ASV_000528 | Myxococcales | II | 0.09 |
| ASV_000188 | Desulfobulbaceae | IV | 0.09 |
| ASV_000255 | Alteromonadales | IV | 0.09 |
| ASV_000482 | Myxococcales | II | 0.08 |
| ASV_000573 | Acidimicrobiales | III | 0.08 |
| ASV_000254 | Acidimicrobiia | III | 0.08 |
| ASV_000342 | Desulfobacteraceae | IV | 0.08 |
| ASV_000340 | Bacteroidales | V | 0.08 |
| ASV_000345 | Desulfobacteraceae | IV | 0.08 |
| ASV_000344 | Bacteroidales | IV | 0.08 |
| ASV_000306 | Desulfobulbaceae | II | 0.08 |
| ASV_000458 | Alteromonadales | IV | 0.08 |
| ASV_000334 | Myxococcales | II | 0.08 |
| ASV_000361 | Alteromonadales | IV | 0.08 |
| ASV_000504 | Pirellulaceae | I | 0.08 |
| ASV_000262 | Desulfuromonadaceae | II | 0.08 |
| ASV_000696 | Piscirickettsiaceae | II | 0.08 |
| ASV_000366 | Sulfurimonas | IV | 0.08 |
| ASV_000491 | Hyphomicrobiaceae | II | 0.07 |
| ASV_000809 | Myxococcales | I | 0.07 |
| ASV_000600 | Piscirickettsiaceae | III | 0.07 |
| ASV_000748 | Myxococcales | II | 0.07 |
| ASV_000393 | Desulfobulbaceae | III | 0.07 |
| ASV_000167 | Lutimonas | IV | 0.07 |
| ASV_000735 | Myxococcales | II | 0.07 |
| ASV_000250 | Alteromonadales | IV | 0.07 |
| ASV_000423 | Acidobacteria | III | 0.07 |
| ASV_000786 | Piscirickettsiaceae | III | 0.07 |
| ASV_000556 | Alphaproteobacteria | II | 0.07 |
| ASV_000218 | Helicobacteraceae | IV | 0.07 |
| ASV_000368 | Verrucomicrobia | IV | 0.07 |
| ASV_000591 | Gemmatimonadetes | II | 0.07 |
| ASV_000449 | Acidimicrobiales | II | 0.07 |
| ASV_000414 | Unknown bacteria | IV | 0.07 |
| ASV_000354 | Bacteroidales | IV | 0.07 |
| ASV_000710 | Gammaproteobacteria | II | 0.07 |
| ASV_000725 | Alteromonadales | II | 0.07 |
| **ASV_000105** | **Helicobacteraceae** | **IV** | **0.07** |
| ASV_000207 | Alteromonadales | IV | 0.07 |
| ASV_000763 | Psychromonas | III | 0.07 |
| ASV_000122 | Flavobacteriaceae | IV | 0.07 |
| ASV_000314 | Alteromonadales | IV | 0.07 |
| ASV_000324 | Deltaproteobacteria | III | 0.06 |
| ASV_000749 | Alteromonadales | II | 0.06 |
| ASV_000593 | Deltaproteobacteria | III | 0.06 |
| ASV_000355 | Alteromonadales | IV | 0.06 |
| ASV_000646 | Alteromonadales | II | 0.06 |
| ASV_000586 | Acidobacteria | II | 0.06 |
| ASV_000723 | Alphaproteobacteria | II | 0.06 |
| ASV_000082 | Desulfobulbaceae | IV | 0.06 |
| ASV_000721 | Psychromonas | IV | 0.06 |
| ASV_000985 | Nitrospina | I | 0.06 |
| ASV_000866 | Myxococcales | II | 0.06 |
| ASV_000168 | Bacteroidales | III | 0.06 |
| ASV_001190 | Acidimicrobiales | II | 0.06 |
| ASV_000930 | Nitrosomonadaceae | I | 0.06 |
| ASV_000532 | Verrucomicrobia | IV | 0.06 |
| ASV_000708 | Acidimicrobiales | II | 0.06 |
| ASV_000527 | Myxococcales | III | 0.06 |
| ASV_001204 | Piscirickettsiaceae | II | 0.06 |
| ASV_001120 | Acidobacteria | II | 0.06 |
| ASV_000443 | Chromatiales | III | 0.06 |
| ASV_000521 | Acidomicrobiales | II | 0.06 |
| ASV_000990 | Flammeovirgaceae | II | 0.06 |
| ASV_000898 | Piscirickettsiaceae | III | 0.06 |
| ASV_000351 | Anaerolineae | II | 0.06 |
| ASV_001010 | Piscirickettsiaceae | II | 0.06 |
| ASV_000799 | Alteromonadales | III | 0.06 |
| ASV_000874 | Deltaproteobacteria | II | 0.05 |
| ASV_000716 | Desulfobulbaceae | II | 0.05 |
| ASV_000533 | Sulfurimonas | IV | 0.05 |
| ASV_000782 | Piscirickettsiaceae | III | 0.05 |
| ASV_000499 | Desulfobulbaceae | IV | 0.05 |

1. Scotland

| ASV | Taxa name | Eco-group | Relative abundance |
| --- | --- | --- | --- |
| **ASV_000008** | ***Psychrilyobacter*** | **IV** | **2.81** |
| **ASV_000014** | **Helicobacteraceae** | **IV** | **1.53** |
| ASV_000004 | Planococcaceae | III | 1.38 |
| ASV_000003 | Alteromonadales | IV | 1.12 |
| ASV_000013 | Helicobacteraceae | IV | 0.70 |
| ASV_000017 | Bacillales | III | 0.67 |
| ASV_000018 | Acidimicrobiales | IV | 0.62 |
| ASV_000060 | Alteromonadales | IV | 0.47 |
| **ASV_000079** | **Alteromonadales** | **IV** | **0.44** |
| **ASV_000078** | **Helicobacteraceae** | **IV** | **0.43** |
| ASV_000016 | Helicobacteraceae | IV | 0.40 |
| ASV_000028 | Acidimicrobiales | III | 0.38 |
| **ASV_000064** | ***Lutimonas*** | **IV** | **0.34** |
| ASV_000026 | Acidimicrobiales | IV | 0.30 |
| ASV_000053 | *Lutimonas* | IV | 0.30 |
| **ASV_000067** | **Helicobacteraceae** | **IV** | **0.27** |
| ASV_000092 | Actinomycetales | IV | 0.25 |
| ASV_000051 | Helicobacteraceae | IV | 0.25 |
| ASV_000023 | Helicobacteraceae | IV | 0.22 |
| ASV_000059 | Desulfobulbaceae | IV | 0.21 |
| ASV_000024 | Alteromonadales | IV | 0.21 |
| ASV_000082 | Desulfobulbaceae | IV | 0.18 |
| ASV_000087 | Helicobacteraceae | IV | 0.17 |
| ASV_000096 | Helicobacteraceae | IV | 0.17 |
| ASV_000175 | Desulfobulbaceae | IV | 0.17 |
| **ASV_000154** | ***Lutimonas*** | **IV** | **0.17** |
| ASV_000044 | Flavobacteriaceae | IV | 0.16 |
| ASV_000050 | Helicobacteraceae | IV | 0.16 |
| **ASV_000039** | **Bacteroidales** | **IV** | **0.15** |
| ASV_000191 | Helicobacteraceae | IV | 0.12 |
| ASV_000301 | Anaerolineae | IV | 0.12 |
| ASV_000350 | Chromatiales | IV | 0.12 |
| ASV_000091 | Desulfobacteraceae | IV | 0.10 |
| ASV_000098 | Caldilineaceae | IV | 0.10 |
| ASV_000066 | Caldilineaceae | IV | 0.10 |
| ASV_000137 | Helicobacteraceae | III | 0.09 |
| **ASV_000292** | ***Lutimonas*** | **IV** | **0.09** |
| ASV_000239 | Helicobacteraceae | IV | 0.09 |
| ASV_000038 | Thiohalorhabdales | IV | 0.09 |
| ASV_000331 | Chromatiales | IV | 0.09 |
| ASV_000090 | Thiohalorhabdales | III | 0.09 |
| **ASV_000421** | ***Lutimonas*** | **IV** | **0.09** |
| ASV_000127 | *Persicirhabdus* | IV | 0.09 |
| ASV_000468 | Helicobacteraceae | IV | 0.09 |
| ASV_000171 | Alteromonadales | IV | 0.08 |
| ASV_000036 | Bacteroidales | IV | 0.08 |
| ASV_000303 | *Lutimonas* | IV | 0.08 |
| ASV_000031 | Myxococcales | III | 0.08 |
| ASV_000485 | Helicobacteraceae | IV | 0.08 |
| ASV_000215 | Desulfuromonadaceae | III | 0.08 |
| **ASV_000107** | **Bacteroidales** | **IV** | **0.07** |
| ASV_000473 | Helicobacteraceae | IV | 0.07 |
| ASV_000356 | Myxococcales | II | 0.07 |
| ASV_000122 | Flavobacteriaceae | III | 0.07 |
| ASV_000110 | Unknown bacteria | III | 0.07 |
| ASV_000401 | *Lutimonas* | V | 0.07 |
| ASV_000415 | Flavobacteriaceae | IV | 0.06 |
| ASV_000120 | *Lutimonas* | II | 0.06 |
| ASV_000300 | Desulfobulbaceae | I | 0.06 |
| ASV_000200 | Acidimicrobiales | III | 0.06 |
| ASV_000262 | Desulfuromonadaceae | II | 0.06 |
| ASV_000041 | Bacteroidales | IV | 0.06 |
| ASV_000582 | Chromatiales | IV | 0.06 |
| ASV_000781 | Chromatiales | IV | 0.06 |
| ASV_000306 | Desulfobulbaceae | I | 0.06 |
| ASV_000180 | Thiotrichaceae | IV | 0.05 |
| **ASV_000114** | ***Desulfococcus*** | **II** | **0.05** |
| ASV_000630 | Thiotrichales | IV | 0.05 |
| ASV_000111 | Actinomycetales | IV | 0.05 |
| ASV_000080 | *Desulfosarcina* | IV | 0.05 |
| ASV_000566 | Chromatiales | III | 0.05 |
| ASV_000168 | Bacteroidales | IV | 0.05 |
| ASV_000641 | Flavobacteriaceae | IV | 0.05 |
| ASV_000086 | Bacteroidales | IV | 0.05 |
| ASV_000247 | Desulfobulbaceae | I | 0.05 |
| ASV_000699 | Chromatiales | IV | 0.05 |
| ASV_000412 | *Persicirhabdus* | IV | 0.05 |
| ASV_000660 | Acidimicrobiales | IV | 0.04 |
| ASV_000552 | Caldilineaceae | IV | 0.04 |
