# Supplementary material for: Comparing quantile regression spline analyses and supervised machine learning for environmental quality assessment at coastal marine aquaculture installations
Source: PeerJ. 2023 Jun 13;11:e15425. doi: 10.7717/peerj.15425 (PMC10274583; doi:10.7717/peerj.15425)
Supplement: Supplemental Information 7 — With red are the values that differ ≤0.1 from the 0.64 good/moderate boundary. [file peerj-11-15425-s007.docx]

**Table S3** Erroneously predicted samples by Quantile Regression Splines (QRS), Random Forest (RF) and both methods (RF+QRS) and the difference between the IQI_MA_ 0.64-good/moderate boundary and the sample’s classified IQI_MA_ for a) Norway and b) Scotland salmon farms. With red are the values that differ ≤ 0.1 from the 0.64 good/moderate boundary.

1. Norway

| **QRS+RF wrong predictions** | IQI_MA_ | IQI_MA_ difference from threshold | IQI_QRS_ | IQI_RF_ |
| --- | --- | --- | --- | --- |
| HJ_588_RepA | 0.73 | 0.09 | 0.54 | 0.41 |
| HJ_588_RepB | 0.73 | 0.09 | 0.55 | 0.46 |
| NK_140_G2_RepB | 0.67 | 0.03 | 0.37 | 0.46 |
| NK_640_G2_RepC | 0.66 | 0.02 | 0.49 | 0.58 |
| ST_68_G1_RepB | 0.42 | 0.22 | 0.74 | 0.70 |

| **QRS-wrong predictions** | IQI_MA_ | IQI_MA_ difference from threshold | IQI_QRS_ |
| --- | --- | --- | --- |
| AK_630_G1_RepA | 0.74 | 0.1 | 0.58 |
| BJ_280_G2_RepA | 0.72 | 0.08 | 0.63 |
| BV_1110_G2_RepC | 0.73 | 0.09 | 0.60 |
| BV_430_G1_RepB | 0.73 | 0.09 | 0.61 |
| BV_430_G2_RepB | 0.74 | 0.1 | 0.60 |
| HJ_50_RepB | 0.1 | 0.54 | 0.66 |
| KA_290_RepA | 0.61 | 0.03 | 0.68 |
| KA_290_RepB | 0.61 | 0.03 | 0.74 |
| NK_640_G2_RepA | 0.66 | 0.02 | 0.56 |

| **Rf-wrong predictions** | IQI_MA_ | IQI_MA_ difference from threshold | IQI_RF_ |
| --- | --- | --- | --- |
| BV_430_G1_RepC | 0.73 | 0.09 | 0.58 |
| BV_430_G2_RepC | 0.74 | 0.1 | 0.55 |
| HJ_488_RepA | 0.68 | 0.04 | 0.51 |
| HJ_488_RepB | 0.68 | 0.04 | 0.60 |
| KA_428_RepA | 0.7 | 0.06 | 0.63 |
| KA_428_RepB | 0.7 | 0.06 | 0.64 |
| KA_504_RepB | 0.64 | 0 | 0.60 |
| NK_140_G1_RepB | 0.67 | 0.03 | 0.56 |
| NK_140_G1_RepC | 0.67 | 0.03 | 0.64 |
| ST_68_G1_RepA | 0.42 | 0.22 | 0.70 |
| ST_68_G1_RepC | 0.42 | 0.22 | 0.69 |
| ST_68_G2_RepB | 0.7 | 0.06 | 0.63 |

1. Scotland

| **QRS+RF wrong predictions** | IQI_MA_ | IQI_MA_ difference from threshold | IQIQRS | IQIRF |
| --- | --- | --- | --- | --- |
| S01_50NE_RepA | 0.79 | 0.15 | 0.56 | 0.45 |
| S05_25S_RepA | 0.61 | 0.03 | 0.64 | 0.67 |
| S05_50N_RepB | 0.67 | 0.03 | 0.56 | 0.52 |
| S06_Start2_RepA | 0.64 | 0 | 0.45 | 0.32 |
| S07_100SE_RepB | 0.75 | 0.11 | 0.60 | 0.61 |

| **QRS-wrong predictions** | IQI_MA_ | IQI_MA_ difference from threshold | IQIQRS |
| --- | --- | --- | --- |
| S01_Ref2_RepB | 0.85 | 0.21 | 0.63 |
| S05_64_RepA | 0.51 | 0.13 | 0.64 |
| S07_57_RepB | 0.65 | 0.01 | 0.58 |
| S07_Ref1_RepA | 0.79 | 0.15 | 0.56 |
| S07_Ref2_RepA | 0.76 | 0.12 | 0.60 |
| S07_Ref2_RepB | 0.77 | 0.13 | 0.59 |

| **Rf-wrong predictions** | IQI_MA_ | IQI_MA_ difference from threshold | IQIRF |
| --- | --- | --- | --- |
| S01_50SE_RepA | 0.33 | 0.31 | 0.66 |
| S05_50S_RepA | 0.69 | 0.05 | 0.63 |
